# Ramulus mori (Sangzhi) alkaloids regulates gut microbiota disorder and its metabolism profiles in obese mice induced by a high-fat diet

**DOI:** 10.3389/fphar.2023.1166635

**Published:** 2023-03-31

**Authors:** Dongdong Liu, Jun Ye, Yu Yan, Yanmin Chen, Hongliang Wang, Mo Wang, Yu Feng, Renjie Li, Xiaoyan Xu, Yu Jiang, Chunfang Lian, Yanfang Yang, Yingying Meng, Yuling Liu, Weizhe Jiang

**Affiliations:** ^1^ College of Pharmacy, Guangxi Medical University, Nanning, China; ^2^ State Key Laboratory of Bioactive Substance and Function of Natural Medicines, Institute of Materia Medica, Chinese Academy of Medical Sciences & Peking Union Medical College, Beijing, China; ^3^ Beijing Wehand-Bio Pharmaceutical Co., Ltd., Beijing, China

**Keywords:** Ramulus mori (Sangzhi) alkaloids, obesity, metabolic syndrome, gut microbiota, lipid metabolism

## Abstract

The imbalance of gut microbiota has been confirmed to have a close pathological and physiological correlation with obesity and metabolic syndrome. Ramulus Mori (Sangzhi) Alkaloids (SZ-A) derived from twigs of mulberry was approved by the National Medical Products Administration of China in 2020 for the treatment of type 2 diabetes mellitus. In addition to its hypoglycemic effect, previous studies have confirmed that SZ-A also alleviates high-fat diet-induced obesity and non-alcoholic fatty liver disease and ameliorates obesity-linked adipose tissue metabolism and inflammation, indicating the potential of SZ-A to regulate obesity and metabolic syndrome. However, whether SZ-A can improve obesity and metabolic syndrome by regulating gut microbiota and its metabolism profiles remains unclear. The purpose of this study was to assess the effect of SZ-A on gut microbiota in obese mice and to explore the association among changes in gut microbiota, obesity, and lipid metabolism. The results showed that oral administration of SZ-A could significantly reduce body weight, fat mass, and the level of total cholesterol and low-density lipoprotein in serum in obese mice induced by a high-fat diet. Interestingly, SZ-A also regulated gut microbiota and changed the fecal metabolite composition of obese mice. Compared with the high-fat diet group, the ratio of Firmicutes to *Bacteroides* changed at the phylum level and the abundance of Bifidobacterium and Akkermansia muciniphila significantly increased at the genus level in the SZ-A group. The gut microbiota of the SZ-A group was reshaped and the relative abundance of microbial genes in bile acid metabolism and fatty acid metabolism were altered, which was consistent with the metabolomics results. Additionally, SZ-A greatly enriched the number of goblet cells and reduced inflammatory colon injury and pro-inflammatory macrophage infiltration induced by a high-fat diet in obese mice. In conclusion, SZ-A can alleviate obesity and metabolic syndrome by improving the gut microbiota and its metabolism profiles of obese mice induced by a high-fat diet.

## 1 Introduction

Metabolic syndrome is a pathological state in which proteins, fats, carbohydrates, and other substances in the human body are metabolically disturbed. It comprises a group of complex metabolic disorder syndrome. As a chronic metabolic disease, obesity, the main driving factor of metabolic syndrome, has become a global epidemic (Bovolini et al., 2021). The average body mass index of the global population is increasing gradually (Bovolini et al., 2021). Between 1980 and 2019, there was a clear increase in the prevalence of obesity, from 3.2% to 12.2% for men and from 6% to 15.7% for women. According to obesity data released by the World Health Organization, there are about 2 billion adults who are overweight, whilst 650 million are obese. By 2025, 2.7 billion adults are expected to be overweight and more than 1 billion will be obese (Boutari and Mantzoros, 2022). In the past, obesity was thought to be the result of a combination of a high-fat diet and lack of exercise based on host genetic characteristics (González-Muniesa et al., 2017). In recent years, studies have found that obesity has an infectivity related to some environmental factors, in addition to genetic factors. An independent risk factor for obesity is the disorder of gut microbiota. Gut microbiota is an important environmental factor that participates in the occurrence and development of metabolic diseases, such as obesity, and plays an important role in regulating energy homeostasis (Turnbaugh and Gordon, 2009; Kobyliak et al., 2016; Dabke et al., 2019; Boutari and Mantzoros, 2022). The imbalance of gut microbiota has been confirmed to have a close pathological and physiological correlation with obesity and metabolic syndrome. Akkermansia muciniphila (Akk) and Bifidobacterium are among the few dominant bacteria that have been proven to be closely related to the development of obesity (Waldram et al., 2009; Ottman et al., 2017). Akk is the only gram-negative trademark verrucous bacterium that is widespread in the mucosa of the human intestine. Previous studies confirmed that oral administration of Akk can reverse high-fat diet-induced obesity in mice by regulating fat cell metabolism and intestinal barrier function without affecting food intake (Cani and de Vos, 2017). In addition, the enrichment of Akk also promoted the richness of the gut microbiota (Derrien et al., 2008), including Firmicutes, Bacteroidetes, and Actinobacteria. Akk can form alliances with Prevotella, Bacteroidetes, Firmicutes, and *Lactobacillus* to maintain the stability of the gut microbiota, and regulate severe disorders of gut microbiota caused by obesity, and disorders of body metabolism (Cani et al., 2007; Yoon et al., 2021).

Ramulus Mori (Sangzhi) Alkaloids (SZ-A) is a group of polyhydroxy-alkaloid effective components extracted and isolated from the mulberry branch, a traditional Chinese medicine, which has been approved by the National Medical Products Administration of China (NMPA) for the treatment of type 2 diabetes mellitus (Approval No.: Z20200002). SZ-A accounts for more than 50% of the total mulberry twig extract, which is majorly composed of 1-deoxynojirimycin (DNJ); fagomine (FA); and 1,4-dideoxy-1,4-imino-D-arabinitol (DAB) (Yang et al., 2017; Liu et al., 2019; Liu et al., 2021; Qu et al., 2021; Zhihua et al., 2023). Among these three major alkaloid components, DNJ has the highest proportion, accounting for more than 50% of the total alkaloid, and the sum of the three main components accounts for more than 80% of the total alkaloid. Besides its good hypoglycemic effect, previous studies have confirmed that SZ-A has multiple pharmacological effects, including alleviating high-fat diet-induced obesity and non-alcoholic fatty liver disease and ameliorating obesity-linked adipose tissue metabolism and inflammation, indicating the potential of SZ-A to regulate obesity and metabolic syndrome (Chen et al., 2022; Sun et al., 2022). Accumulated evidence confirmed that gut microbiota and their metabolic profiles played an important role in the occurrence and development of obesity and metabolic syndrome. However, whether SZ-A can improve obesity and metabolic syndrome by regulating gut microbiota and its metabolism profiles remains unclear.

**SCHEME 1:**
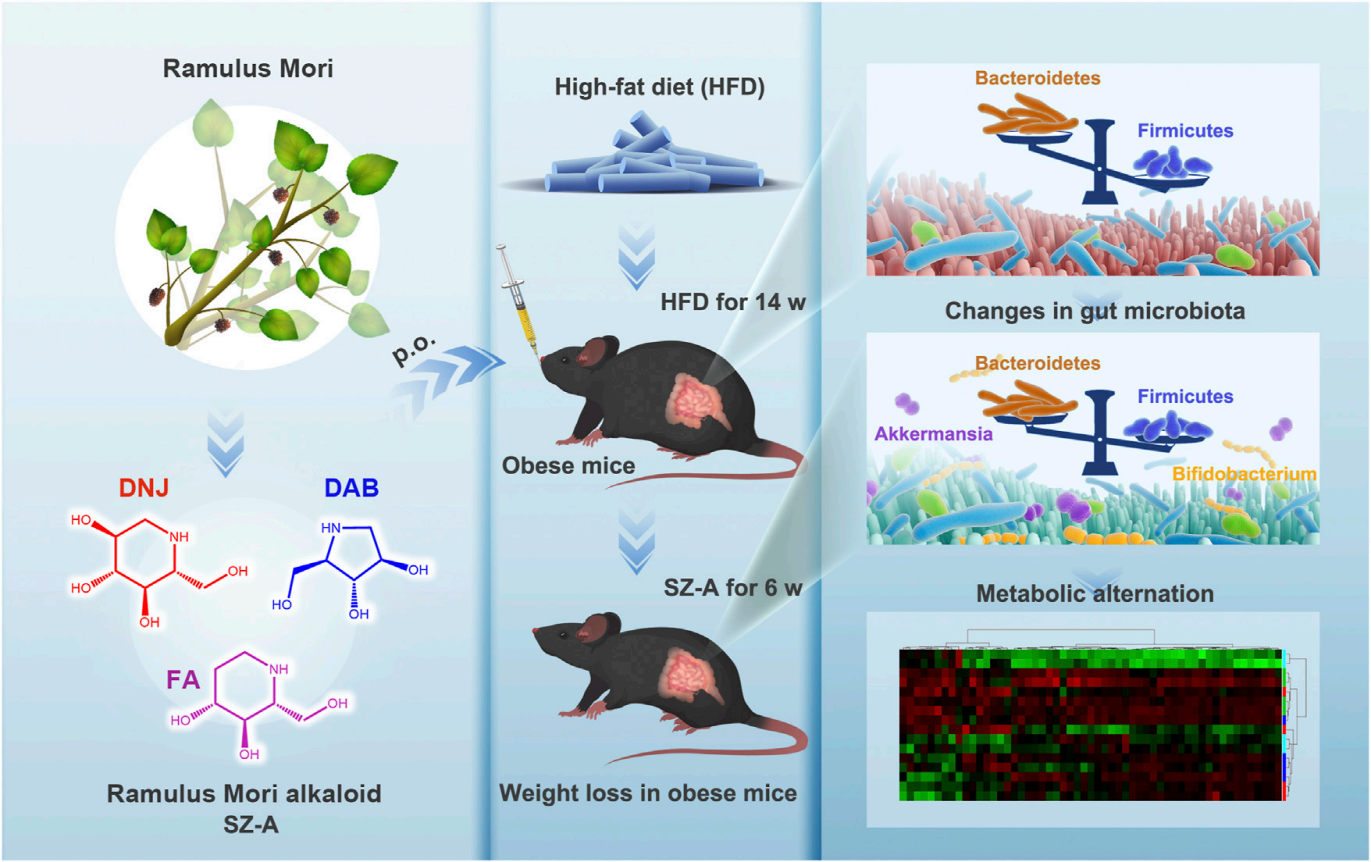
Schematic illustration of Ramulus Mori (Sangzhi) Alkaloids (SZ-A) regulating gut microbiota disorder and its metabolism profiles in high-fat diet-induced obese mice.

In the present study, we aimed to explore the role of gut microbiota and its metabolic profiles in the improvement of obesity and metabolic syndrome by SZ-A. We used obese mice induced by a high-fat diet (HFD) as a model and investigated the effects of SZ-A on reducing body weight and improving metabolic syndrome in obese mice after treatment. The changes in gut microbiota and the metabolic profiles of obese mice were determined by 16s amplicon sequencing and targeted metabolomics (Scheme 1). To the best of our knowledge, this is the first study to investigate the regulation of gut microbiota and its metabolism in HFD-induced obese mice. The results of the present study provide insights into the improvement of obesity and metabolic syndrome by SZ-A.

## 2 Materials and methods

### 2.1 Materials

SZ-A powder (Lot Number: J202012017, the total polyhydroxy alkaloids content in SZ-A powder is 57.0%, which includes 36.95% DNJ, 7.09% FA, and 7.79% DAB) was a kind gift from Beijing Wehand-Bio Pharmaceutical Co., Ltd. (Beijing, China). The rodent diet with 60% kcal% fat was purchased from Research Diets Inc. (Product No.: D12492; New Brunswick, NJ, United States of America).

### 2.2 Design of animal experiments

C57BL/6J (6 to 8-week-old, male) were obtained from Beijing Huafukang Biotechnology Co., Ltd. (Beijing, China). All animal care and experimental procedures were approved by the Beijing Experimental Animal Research Center (Application Date: 15/10/2020, Ethics No.: 2020001). All mice were reared at 24°C with a 12-h light/dark cycle. After 1 week of adaptation, the mice were randomly divided into five groups: normal control group (NC; *n* = 10), high-fat diet group (HFD; *n* = 10), SZ-A treatment group (SZ-A, 200 mg/kg; *n* = 10), metformin treatment group (Met, 300 mg/kg; *n* = 10), and acarbose treatment group (Acb, 100 mg/kg; *n* = 10). Mice in the NC group were given a maintenance diet, and mice in other groups were fed a high-fat diet with 60% kcal% fat for 14 weeks. Mice in the HFD group were then administered normal saline, SZ-A 200 mg/kg/d, Met 300 mg/kg/d, and Acb 100 mg/kg/d for 6 weeks. After 6 weeks of SZ-A administration, the fat content of mice was detected by the Faxitron Ultrafocus instrument. Muscle anesthesia was performed on mice with Zoletil^®^ (Virbac, Cedex, France). The mice were placed in the prone position for X-ray scanning and the fat content was calculated. Body weight was measured weekly during the experiment, and mouse feces were collected and placed in a 2 mL cryo-storage tube in liquid nitrogen 1 d before the end of the experiment and then stored at −80°C until analysis. Mice fasted overnight before euthanasia, and subcutaneous fat and abdominal fat tissue were collected and weighed. Colon tissue was collected and fixed in 4% paraformaldehyde solution and placed at room temperature for subsequent analysis.

### 2.3 Histopathological analysis of colon

To observe pathological changes in colon tissue obtained from each experimental group, approximately 2 cm of colon tissue was fixed in 4% paraformaldehyde, then 5 μm paraffin sections were prepared, stained with hematoxylin and eosin (H&E), and inflammatory changes, inflammatory and crypt damage, and goblet cell numbers were evaluated by optical microscopy.

### 2.4 Analysis of gut microbiota composition

#### 2.4.1 Genomic DNA extraction and PCR amplification

By using the CTAB method, the genomic DNA of the samples was extracted, and DNA purity and concentration were determined using 1% agarose gel. The obtained sample of DNA was placed in an EP tube and then diluted with sterile water to 1 ng/μL.

Amplification of genomic DNA was conducted using diluted genomic DNA. This operation used Phusion^®^ High-Fidelity PCR Master Mix with GC Buffer, high-efficiency high-fidelity enzyme (New England Biolabs), and specific primers with Barcode to improve the amplification efficiency and accuracy.

#### 2.4.2 Mixing and purification of PCR products

PCR products were detected by electrophoresis using a 2% concentration of agarose gel. Following full mixing, the PCR products were detected by 2% agarose gel electrophoresis using the same amount of PCR products according to their concentration. The product was recovered from the target strip using the Qiagen Gel Extraction Kit (Qiagen, Germany).

#### 2.4.3 Library construction and sequencing

Sequencing libraries were constructed with the TruSeq^®^ DNA PCR-Free Sample Preparation Kit (Illumina) according to the instruction for use. The index codes were updated. The constructed library was quantified by Qubit^@^ 2.0 Fluorometer (ThermoScientific) and Q-PCR. After the qualified library, NovaSeq6000 was used for sequencing.

#### 2.4.4 Data analysis

Separating the sample data from the disembarkation data was based on the Barcode sequence and the PCR-amplified primer sequence. Each sample’s reads were spliced with FLASH after the Barcode and primer sequences were removed. The Raw Tags sequence was derived from this splicing sequence. The Raw Tags obtained by splicing need to be processed with strict filtering to obtain high-quality Tags data (Clean Tags). Tags are processed concerning Qiime’s Tags quality control process, and then the chimera sequence is removed. As a result of comparing the Tags sequence with the species annotation database, the chimera sequences are detected, and finally, the chimera sequences are removed to obtain the Effective Tags.

To cluster all Effective Tags of every sample, the Uparse algorithm is used, which clusters sequences into Operational Taxonomic Units (OTUs) with 97% consistency. In parallel, representative sequences of OTUs were chosen. A representative sequence of OTUs is chosen according to the algorithm principle based on the sequence with the highest frequency among OTUs. To obtain taxonomic information, the Mothur method and SSUrRNA database of SILVA132 (with a threshold of 0.8–1) was used to primarily identify species of OTUs. Each sample was analyzed at every taxonomic level, such as kingdom, phylum, class, order, family, genus, and species. The phylogenetic relationships of all OTUs representative sequences were obtained by fast multiple sequence alignments using MUSCLE software. Lastly, the homogenized data of each sample are compared, and the sample with the smallest amount of data is used as the standard. Following the homogenization treatment, Alpha diversity analysis and Beta diversity analysis are performed.

### 2.5 Untargeted metabolomics

#### 2.5.1 Metabolite extraction

Tissue samples (100 mg) obtained from liquid nitrogen grinding were placed in EP tubes, and 500 μL of 80% methanol aqueous solution was added. After vortexing and shaking, the samples were left in an ice bath for 5 min, and then centrifuged at 15,000 *g* and 4°C for 20 min. The appropriate amount of supernatant was diluted with water to 53% methanol. After centrifugation at 15,000 *g* for 20 min at 4°C, the supernatant was collected and injected into the sample for analysis by LC-MS.

#### 2.5.2 UHPLC-MS/MS analysis metabolite identification

A Vanquish UHPLC system was coupled to an Orbitrap Q ExactiveTM HF mass spectrometer for UHPLC-MS/MS analysis. Using CD 3.1 database search software, the offline data files were imported, and the mass-to-charge ratios, retention times, and other parameters of each metabolite were simply screened. The mass deviation was set to 5 ppm and the retention time deviation was set to 0.2 min. To increase the accuracy of the identification, the peaks of different samples were aligned. For peak extraction, we set a signal-to-noise ratio of 3, a signal intensity deviation of 30%, a minimum signal intensity, a mass deviation of 5 ppm, plus ion, and other information, while the peak area was quantified, and then the target ion was integrated. According to molecular ion peaks and fragment ions, the molecular formula was predicted and compared with databases from mzCloud, mzVault, and Masslist. In addition to removing background ions, original quantitative results were standardized, and metabolite identification and relative quantitative results were obtained.

#### 2.5.3 Data analysis

The identified metabolites were annotated using the KEGG database. Multivariate statistical analysis was performed using metaX, a metabolomics data processing software, to transform the data and perform partial least square discriminant analysis (PLS-DA), and then obtain the VIP value of each metabolite. Between the two groups, univariate analysis was based on a *t*-test to calculate the statistical significance (*p* value) and the fold change (FC value) of each metabolite. The default standard for differential metabolite screening was VIP >1, *p* value <0.05, and FC ≥ 2 or FC ≤ 0.5. A volcano map was created using the R package ggplot2. The parameters, including the VIP value, log2 (FoldChange), and -log10 (*p* value) of metabolites, were integrated to screen the target metabolites.

### 2.6 Targeted metabolomics

#### 2.6.1 Metabolite extraction

The sample was placed in an EP tube containing 100 mg and vortexed fully with 800 μL cold methanol/acetonitrile/water (2:2:1,v/v/v) to extract metabolites. An isotope internal standard solution was added to the extraction solvent for the absolute quantification of metabolites. The samples were shaken for 2 min at 4°C, incubated on ice for 20 min, and centrifuged at 14,000 *g* for 20 min at 4°C. After collecting the supernatant, the samples were filtered through a 96-well protein precipitation plate. The eluate was collected and dried by vacuum at 4°C. Then, the samples were redissolved in 100 μL acetonitrile/water (1:1,v/v) and centrifuged at 14,000 *g* for 15 min at 4°C, and the supernatant was collected for LC-MS analysis.

#### 2.6.2 Measurement parameters

The elution was carried out with gradients of 15% A (0–1 min), 20% A (3–4 min), 30% A (6 min), 50% A (10–15.5 min), and 15% A (15.6–23 min) at a flow rate of 300 μL/min using 90% H2O, 2 mM ammonium formate, and 10% acetonitrile as mobile phase A, and 0.4% methanol formate as mobile phase B. For RPLC separation: the column temperature (40°C) and injection volume (2 μL); mobile phase A (5 mM ammonium acetate and 0.2% NH_3_·H_2_O); mobile phase B (99.5% acetonitrile and 0.5% NH_3_·H_2_O). The elution was carried out at 400 μL/min with gradients of 95% A (0 min), 40% A (5 min), 0% A (11–13 min), and 95% A (13.1–16 min). Mass spectrometry was performed in positive and negative switching modes and quantitative data acquisition was performed in MRM mode. Quality control (QC) samples were placed into sample cohorts throughout the assay to evaluate the stability and repeatability of the assay.

#### 2.6.3 Data analysis

The obtained data were normalized and uploaded to SIMCA-P for multivariate data analysis. Cross-validation and response permutation tests were conducted to assess the robustness of the model. We calculated the variable importance of each variable in the OPLS-DA model to determine its contribution to classification.

### 2.7 Statistical analysis

Statistical analysis was performed by Student’s t-test for two groups or one-way analysis of variance for more than two groups using GraphPad Prism version 7.00 for Windows (GraphPad Software, La Jolla, CA, United States of America). Differences were considered statistically significant at *p* value <0.05.

## 3 Results

### 3.1 SZ-A effectively regulates the body weight of obese mice induced by HFD

**FIGURE 1:**
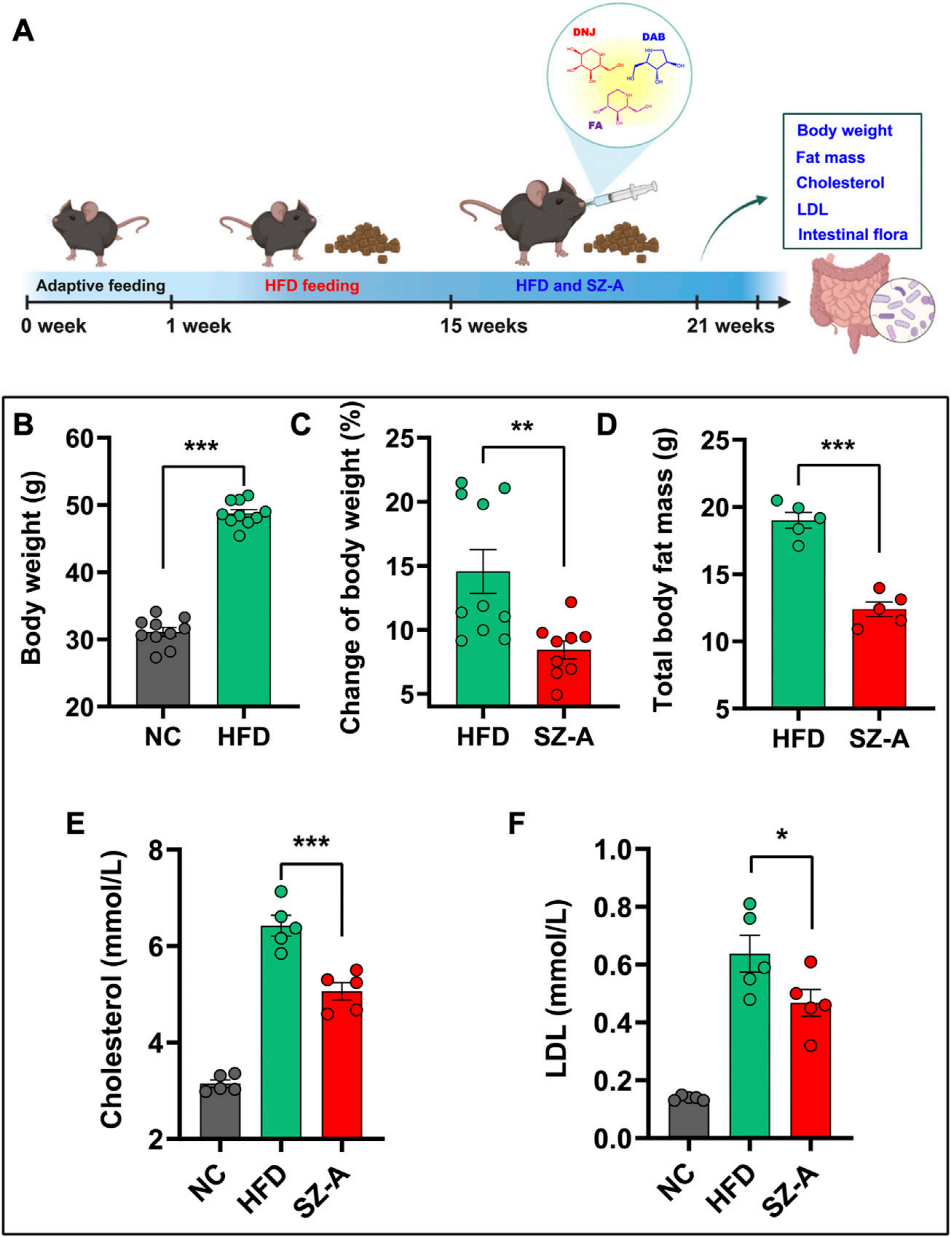
SZ-A slowed weight gain in obese mice induced by HFD. **(A)** The flow chart of relieving weight gain in obese mice by oral administration of SZ-A. In this experiment, C57BL/6J mice were fed a high-fat diet for 14 weeks and then orally administered SZ-A (200 mg/kg/day) daily for 6 weeks. **(B)** The Body weight of mice fed with a maintenance diet (NC) or HFD (*n* = 10). **(C)** The change of body weight of mice treated with SZ-A (200 mg/kg) (*n* = 9–10). **(D)** Total fat mass of mice treated with SZ-A (200 mg/kg) (*n* = 5). **(E)** Effect of SZ-A (200 mg/kg) on serum level of cholesterol (*n* = 5). **(F)** Effect of SZ-A (200 mg/kg) on serum level of LDL (*n* = 5). Each value is expressed as mean ± SEM. ***p < 0.001, **p < 0.01. *p < 0.05.

To investigate the therapeutic effect of SZ-A on obesity, C57BL/6J mice were fed a high-fat diet (HFD) for 14 weeks, and then SZ-A (200 mg/kg/d) was orally administered to mice daily for 6 weeks with continued HFD feeding (Figure 1A). The current clinical hypoglycemic dose is 5 mg/kg, which was converted to 60 mg/kg for mice. To explore the potential of SZ-A in reducing body weight, we previously investigated the weight loss effect of 100 and 200 mg/kg SZ-A treatment for 8 weeks in HFD-feeding KKAy mice and 400 mg/kg SZ-A treatment for 6 weeks in HFD-feeding C57BL/6J mice (Liu et al., 2021; Chen et al., 2022; Sun et al., 2022). The results showed that at this intervention time and dose, SZ-A has a good effect on weight loss. According to the previous results, HFD-feeding C57BL/6J mice were used as a model, and 200 mg/kg SZ-A was used to intervene for 6 weeks to further explore the weight loss effect of SZ-A and its regulatory effect on gut microbiota and metabolism in the present study. As shown in Figure 1B, mice fed an HFD had significantly higher body weight than mice fed a normal diet, and treatment with SZ-A significantly slowed the HFD-induced weight gain as compared with the HFD group (Figure 1C). In addition to the body weight of obese mice, the total body fat mass was also measured using magnetic resonance imaging (MRI). As shown in Figure 1D, the administration of SZ-A reduced the total body fat mass compared with the HFD group. Additionally, the serum total cholesterol and low-density lipoprotein (LDL) levels were significantly reduced after the treatment of SZ-A (Figures 1E, F). The results showed that SZ-A effectively relieved obesity induced by HFD in C57BL/6J mice, which is consistent with our previous research results (Chen et al., 2022).

### 3.2 Changes in gut microbiota composition induced by SZ-A

The above-mentioned results indicated that SZ-A could effectively regulate the body weight of HFD-induced obese mice. Obesity, as a chronic metabolic disease, has been confirmed to be closely related to the gut microbiota, and oral drugs have the most significant impact on gut microbiota and metabolism. To investigate the changes in gut microbiota in HFD-induced obese mice, C57BL/6J mice were first fed an HFD for 14 weeks and received the treatment by oral gavage of SZ-A with continued HFD feeding. After the experiment, the feces of all mice were collected, the V3 -- V4 regions of the 16S rRNA gene were sequenced by Illumina NovaSeq sequencing, and the bacterial community structure was analyzed.

**FIGURE 2:**
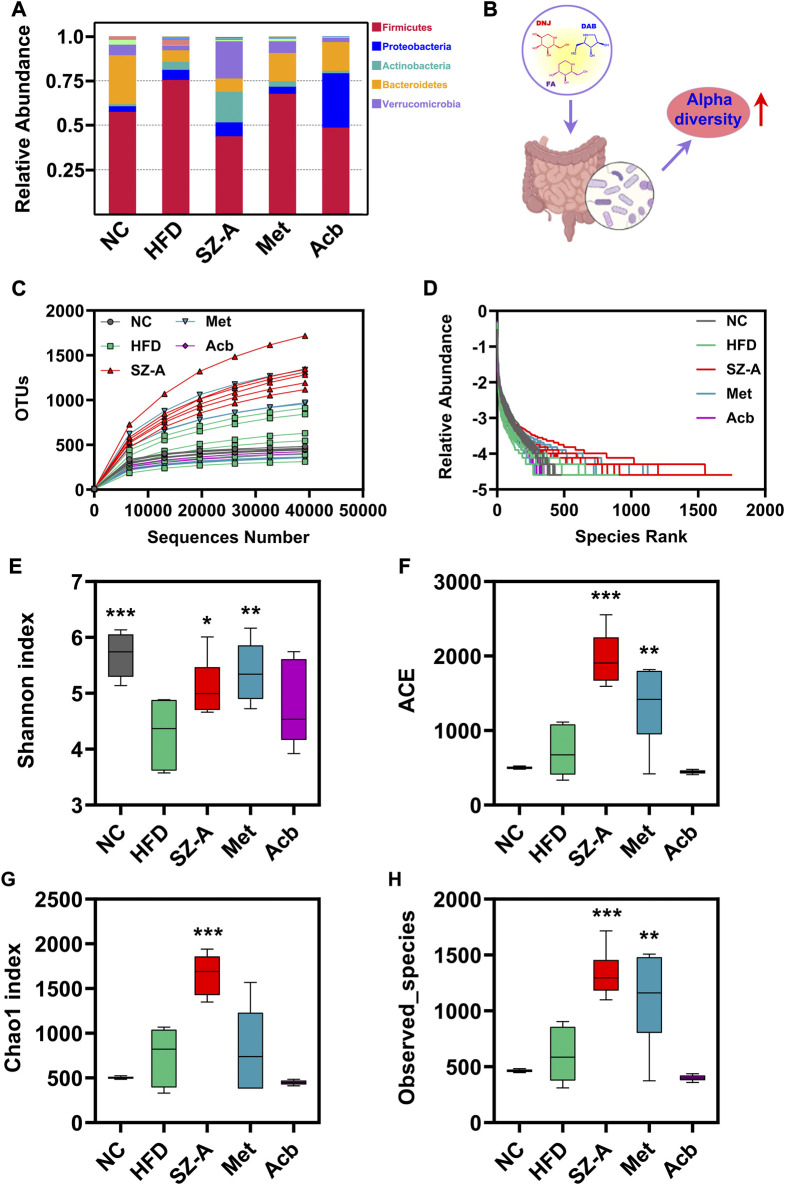
The gut microbiota diversity and richness of HFD-induced obese mice were improved by SZ-A through alpha diversity analysis. **(A)** Relative abundance of species columnar cumulative plot. **(B)** Alpha diversity indices increased after treatment with SZ-A. **(C)** Rarefaction curve. **(D)** Rank abundance curve. **(E–H)** Four alpha diversity indices (Shannon, ACE, Chao1, and Observed_species) were calculated (*n* = 6). The complexity of the sample community could be reflected by the index values. Each value is expressed as mean ± SEM. ***p < 0.001, **p < 0.01. *p < 0.05 compared with HFD.

To study the species composition of each sample, OTUs (Operational Taxonomic Units) clustering was conducted with 97% identity. Next, species annotation was performed on the OTUs sequence. According to the species annotation results, the top 10 species with maximum abundance in each sample or grouping at the phylum level were selected to generate a columnar accumulation diagram of the relative abundance of species, to visually view the species with a high relative abundance and their proportion in each sample at different classification levels (Figure 2A). The results were analyzed for microbial diversity. Alpha diversity reflects the richness and diversity of the microbial community within a sample (Figure 2B). The rarefaction and rank abundance curves (a curve reflecting the rationality of the volume of sequencing data and the richness of species) showed that there was a reasonable amount of sequencing data and a uniform distribution of species in this experiment (Figures 2C, D). Shannon, Chao1, ACE, and observed-species indices (an index for assessing species richness and diversity in a sample) showed that the gut microbiota diversity and richness of obese mice fed an HFD increased significantly after treatment with SZ-A (Figures 2E–H). Except for SZ-A, Met also significantly increased the richness and diversity of gut microbiota in obese mice, as evidenced by the increased Shannon, ACE, and observed-species indices compared with the HFD group. However, no significant changes were observed in the Acb group.

**FIGURE 3:**
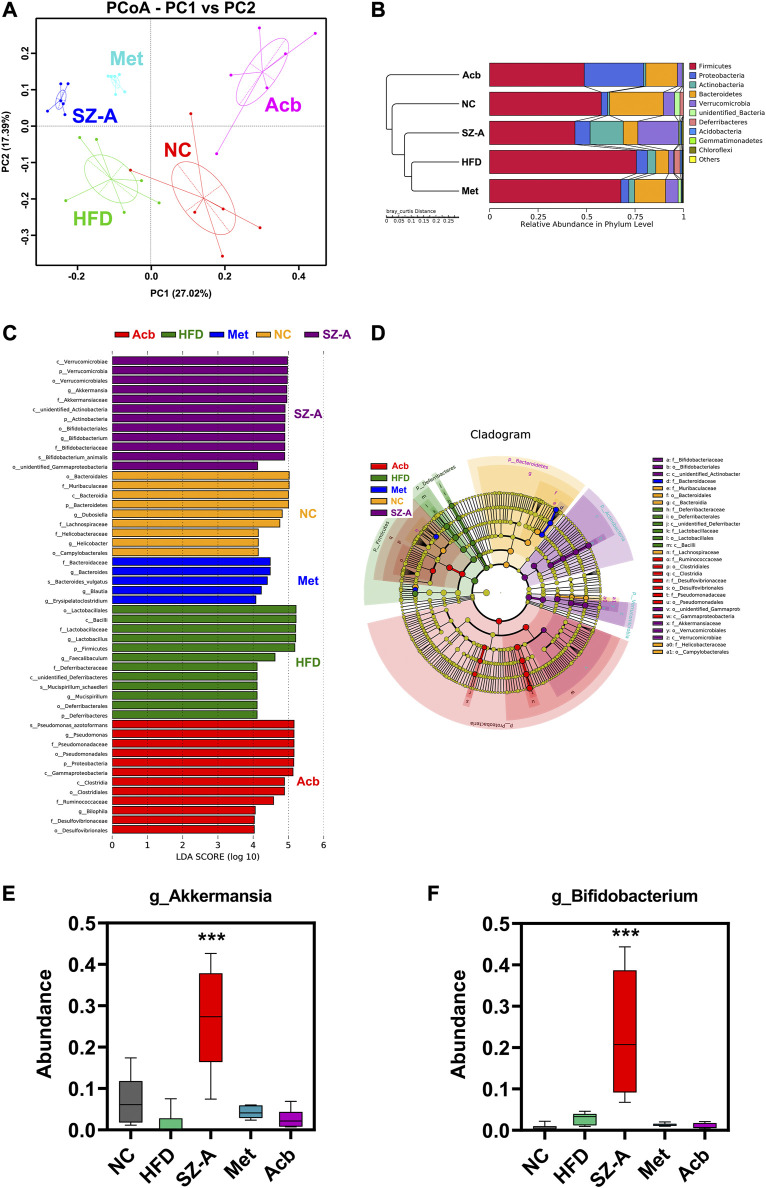
SZ-A altered the gut microbiota structure in HFD-induced obese mice through beta diversity analysis. **(A)** PCoA, Principal Co-ordinates Analysis. **(B)** UPGMA, Unweighted Pair-group Method with Arithmetic Mean. **(C)** LEfSe, LDA Effect Size. **(D)** LEfSe, branching diagram. **(E)** The abundance of Akkermansia muciniphila at the genus level (*n* = 6). **(F)** The abundance of Bifidobacterium at the genus level (*n* = 6). Each value is expressed as mean ± SEM. ***p < 0.001 compared with HFD.

Beta diversity is a comparative analysis of the microbial community composition of different samples to identify the differences between different groups. The results of principal coordinates analysis (PCoA) showed that there was a significant cluster of microbial community composition in the SZ-A group, indicating that SZ-A treatment had a substantial effect on the gut microbiota of HFD-induced obese mice (Figure 3A). To investigate specific changes in the gut microbiota, we performed UPGMA cluster tree analysis to compare the relative abundance of major groups identified in each group sequencing. At the phylum level, the ratio of Firmicutes to Bacteroidetes was severely misaligned in the HFD group as compared with the normal control group (NC): the abundance of Firmicutes increased significantly, whereas that of Bacteroidetes decreased significantly. Interestingly, SZ-A could adjust the ratio of these two bacteria to achieve a flora structure similar to that of the normal control group (NC) (Figure 3B). The ratio of Firmicutes/Bacteroidetes is a sign of the dynamic balance of gut microbiota, and the decrease in the Firmicutes/Bacteroidetes ratio is directly related to weight loss (Grigor’eva, 2020).

To further explore the differences between each sample, Lefse analysis (LDA Scoer = 4) was performed. Compared with the HFD group, the microflora in the SZ-A group showed significant changes, which were characterized by significantly increased levels of Verrucomicrobia and Actinobacteria (Figures 3C, D). At the genus level, Akkermansia muciniphila (Akk) and Bifidobacterium in the SZ-A group were more abundant than those in other groups (Figures 3E, F). Akk has been proven to promote intestinal barrier integrity, regulate immune response, inhibit inflammation, and balance gut microbiota and other functions, which makes it have great potential in the treatment of metabolic diseases (Wang et al., 2020). These results indicated that SZ-A could alter the gut microbiota structure in HFD-induced obese mice.

### 3.3 SZ-A regulates the metabolism of obese mice induced by HFD

**FIGURE 4:**
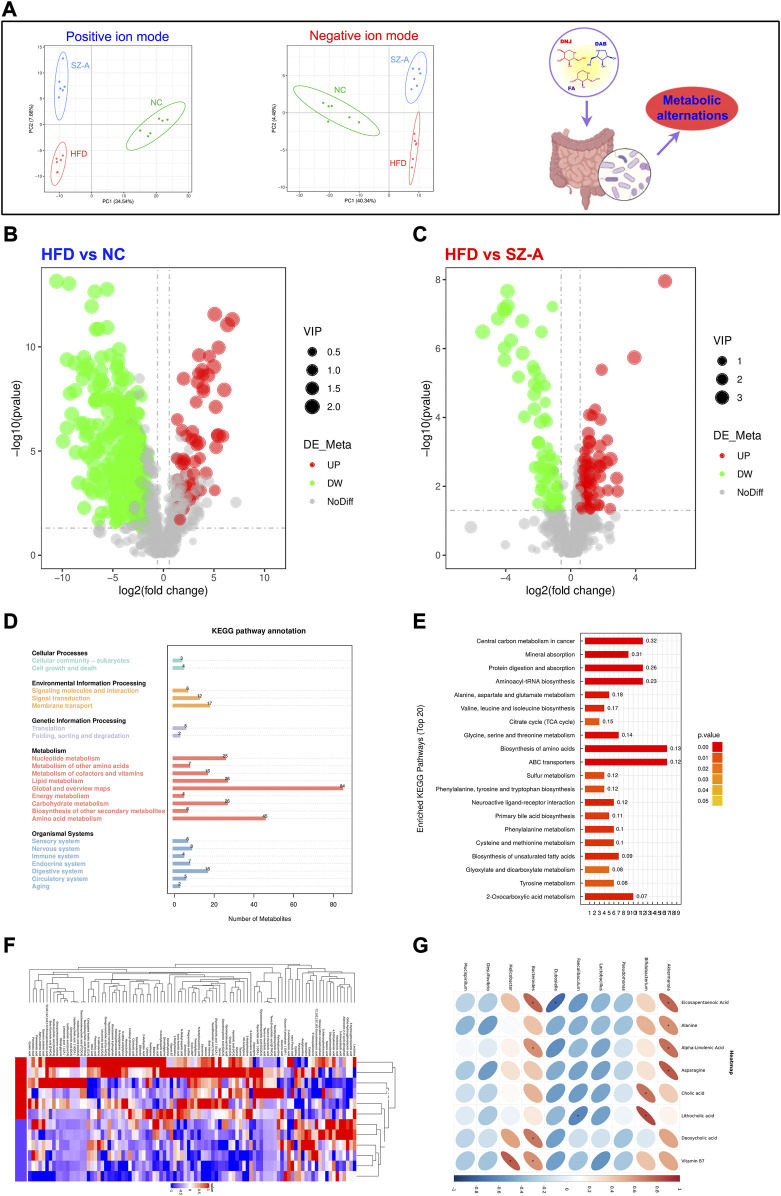
SZ-A regulated metabolic disorders in HFD-induced obese mice. **(A)** PLS-DA, Partial Least Squares Discrimination Analysis. **(B, C)** Volcano plot. **(D)** Kyoto Encyclopedia of Genes and Genomes (KEGG) path annotation. **(E)** KEGG enrichment pathway. **(F)** Heat map. **(G)** The correlation analysis between gut microbiota and host metabolites after the SZ-A intervention.

To better understand the impact of SZ-A on the intestinal microenvironment, metabolomics was used to detect changes in metabolites in mouse feces. Before the end of the experiment, the feces of all mice were collected, and metabolomic studies were conducted using LC-MS/MS. To better study the intervention effect of SZ-A on HFD-induced obese mice, multivariate statistical analysis was used to screen for differential metabolites in mice after SZ-A intervention. The results of Partial Least Squares Discrimination Analysis (PLS-DA) showed that no matter in the positive or negative ion mode, the dispersion degree of samples in each group is small, and the concentration trend is obvious. The samples of the normal control group (NC) and HFD group showed a tendency to separate, which suggested HFD-induced changes in metabolite levels in HFD-induced obese mice. After the treatment of SZ-A, the samples in the SZ-A group were more similar to those in the normal control group (NC), suggesting that SZ-A intervention could regulate the metabolic disorder in HFD-induced obese mice (Figure 4A). In addition, the points of the quality control (QC) samples were concentrated, indicating that the stability and repeatability of the analysis method were good and that the experimental results were reliable.

To further reveal the changes in internal metabolites in obese mice induced by an HFD before and after SZ-A intervention and screen differential metabolites, VIP, FC, and *p*-value were mainly referred to. VIP refers to the Variable Importance in the Projection of the first principal component of the PLS-DA model and represents the contribution of metabolites to the grouping. FC refers to the FoldChange, which is the ratio of the mean values of all biological repeats for each metabolite in the comparison group. The threshold was set as VIP >1.0, FC > 1.2, or FC < 0.833, and a *p* value <0.05. The results showed that the metabolites in the HFD group were significantly different from those in the NC and SZ-A groups (Figures 4B, C). Based on the screened differential metabolites, further KEGG enrichment analysis was conducted, and the results showed that compared with the HFD group, the differential metabolites were mainly enriched in the metabolic pathway after treatment with SZ-A (Figure 4D). Among them, there were significant differences in amino acid, unsaturated fatty acid, and bile acid metabolism between the SZ-A and HFD groups (Figure 4E). The results of the heat map showed that the expression levels of beneficial substances such as eicosapentaenoic acid (EPA), docosatetraenoic acid (DHA), alpha-linolenic acid, and lipocholic acid (LCA) in the process of alleviating obesity and metabolic disorders were significantly increased after treatment with SZ-A in HFD-induced obese mice (Figure 4F). We have performed the correlation analysis between gut microbiota and host metabolites after the SZ-A intervention. The top 10 bacterial groups with relative abundance at the genus level were correlated with targeted metabolomics results. As shown in Figure 4G, Akk and Bifidobacteria were positively correlated with EPA, amino acids, and bile acids, indicating that after the intervention of SZ-A, the increase in the abundance of Akk and bifidobacteria in the intestine was significantly correlated with the improvement of metabolism. One limitation of this study is that the metabolic regulation of SZ-A was not compared with NC group. These results indicated that HFD could induce obesity and lead to metabolic disorders in mice, and SZ-A could effectively regulate metabolic disorders in HFD-induced obese mice.

### 3.4 SZ-A alleviates colon inflammatory injury and pro-inflammatory macrophage infiltration in obese mice induced by HFD

**FIGURE 5:**
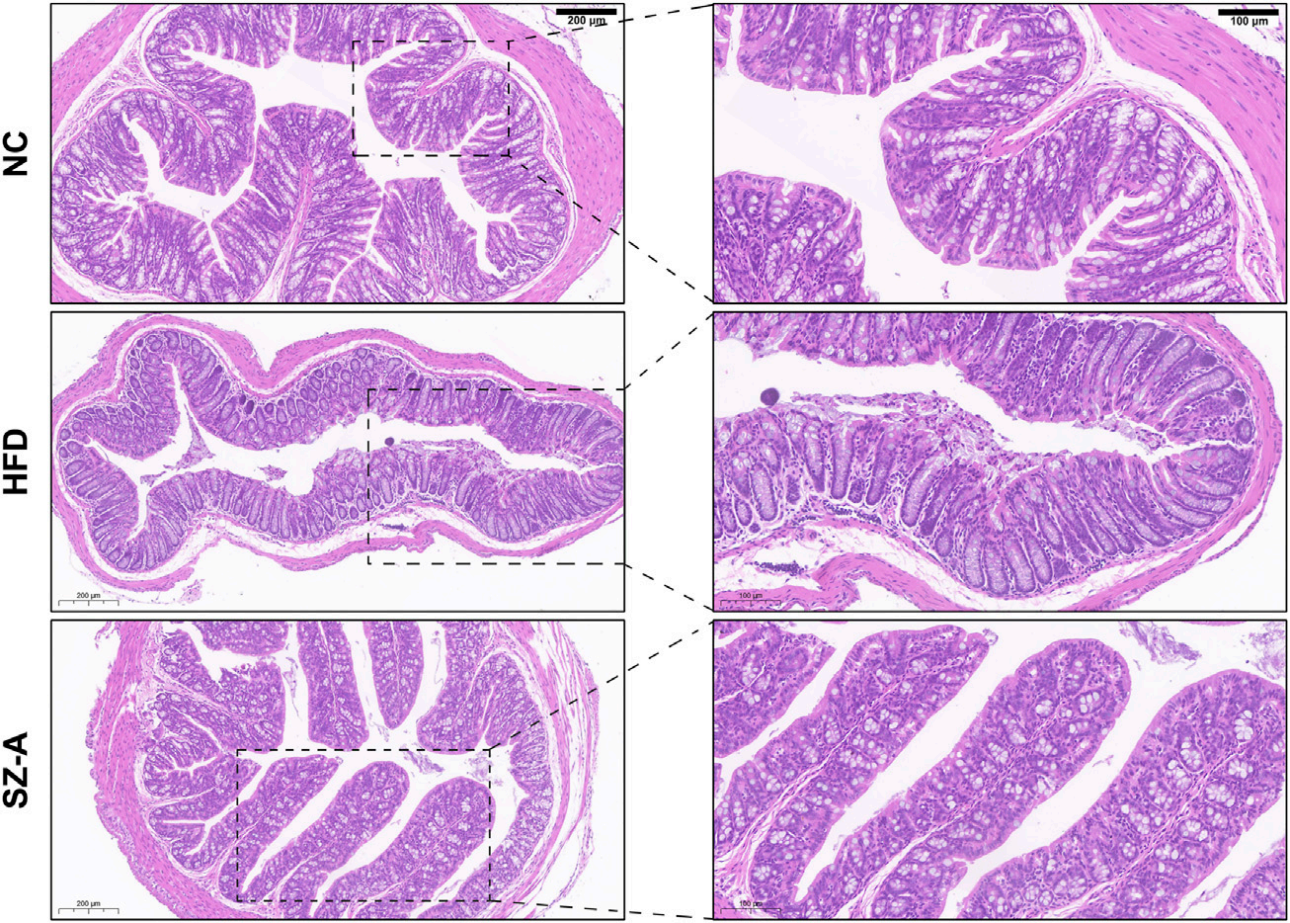
SZ-A alleviated colon inflammatory injury and pro-inflammatory macrophage infiltration in mice. Representative H&E staining images of the colon section.

Considering that obese patients experience chronic low-grade inflammation for a long time, the integrity of the intestinal barrier plays a crucial role in the occurrence and development of obesity. Histological changes in the colon were assessed by hematoxylin and eosin (H&E) staining. As shown in Figure 5, there was inflammatory cell infiltration in the mucosa and submucosa, goblet cells were greatly reduced, and surface epithelium was damaged in the HFD model group. After SZ-A treatment, there was no obvious damage to the mucosal epithelium, no obvious inflammatory infiltration, and abundant goblet cells. It has been suggested that long-term treatment with SZ-A can restore intestinal barrier integrity caused by obesity.

## 4 Discussion

Metabolic syndrome is a pathological state in which proteins, fats, carbohydrates, and other substances in the human body are metabolically disturbed. It comprises a group of complex metabolic disorder syndromes. As a chronic metabolic disease, obesity is not only the main driving factor of metabolic syndrome but also closely related to gut microbiota. The imbalance of gut microbiota has been confirmed to have a close pathological and physiological correlation with obesity and metabolic syndrome. In recent years, the study of gut microbes has turned our traditional concept of living organisms on its head. Many novel research results, such as the gut-brain axis, gut-liver axis, and gut-pancreatic axis, have made it clear to us that the gut microbiota is the second genome besides the genome of our body, constituting the so-called “super-organism” together with the body itself (Zheng et al., 2016; Loomba et al., 2019; Yang et al., 2020). At the same time, these studies have also revealed that gut microbes play a crucial role in maintaining our health. By interacting with the body, gut microbes create a unique “environment” of body-specific superorganisms, thus playing a very important role in the occurrence and development of diseases. Microbial imbalance is a trigger point for many chronic metabolic diseases, including obesity. Restoring the dynamic balance of the gut microbiota is crucial for the treatment of metabolic diseases. Metformin and acarbose are the most commonly used drugs to treat type 2 diabetes mellitus and obesity. It was found that metformin can improve patients’ glycolipid homeostasis by increasing Akkermansia muciniphila and butyrate and propionate producing microbiome (Vallianou et al., 2019). Acarbose can increase the relative abundance of *lactobacillus* and bifidobacterium in gut microbiota and reduce the relative abundance of *bacteroides*, thus changing the relative abundance of microbial genes involved in bile acid metabolism, and producing beneficial effects on metabolism (Gu et al., 2017). Therefore, it is a new and effective treatment method to regulate gut microbiota and restore its dynamic balance, which has great prospects (Carneiro et al., 2022).

SZ-A has been approved by the NMPA of China in 2020 for the treatment of type 2 diabetes mellitus. In addition to its hypoglycemic effect, previous studies have confirmed that SZ-A also alleviates high-fat diet-induced obesity and non-alcoholic fatty liver disease and ameliorates obesity-linked adipose tissue metabolism and inflammation, indicating the potential of SZ-A to regulate obesity and metabolic syndrome (Chen et al., 2022; Sun et al., 2022). However, whether SZ-A can improve obesity and metabolic syndrome by regulating gut microbiota and its metabolism profiles remains unclear. In this study, we evaluated the effect of SZ-A on gut microbiota in HFD-induced obese mice. We found that the composition of the gut microbiota changed significantly after treatment with SZ-A. For example, the relative abundance of Firmicutes with strong energy utilization efficiency was significantly higher in the fecal community of obese mice in the HFD group, whereas the relative abundance of *Bacteroides* with low nutrient absorption efficiency was low. After treatment with SZ-A, the Bacteroidetes to Firmicutes ratio was similar to that in the normal control group (NC). At the genus level, we further observed that the abundance of two genera, Akkermansia muciniphila (Akk) and Bifidobacterium, was significantly increased in the SZ-A group compared with the HFD group. Akk and Bifidobacterium may be involved in reducing inflammation and obesity in HFD-induced obese mice. It has been reported that in mice with obesity/type 2 diabetes, the abundance of Akk decreased dramatically. If the abundance of Akk can be increased by prebiotics or feeding Akk, the metabolic problems caused by an HFD can be ameliorated without dietary changes, and metabolic disorders can even be reversed. Similarly, lower levels of Akk have been confirmed in clinical trials in children and adults with obesity. At the same time, a large number of studies have shown that Akk bacteria can also promote GLP-1 secretion, insulin sensitivity, and intestinal microecological richness, and plays an important role in the treatment of metabolic syndrome (Derrien et al., 2008; Cani and de Vos, 2017; Yoon et al., 2021). Studies have found that Bifidobacterium has bile brine hydrolase activity, which can adjust the size of the bile acid pool and the proportion of each bile acid component, as well as convert primary bile acid and binding bile acid into secondary bile acids, like deoxycholic acid and stone cholic acid. Activation of G protein-coupled receptor 5 (TGR5) and Farnesoid X receptor (FXR) improves metabolism and plays an anti-obesity role (Li et al., 2022).

Lipopolysaccharide (LPS) is the main component of the cell wall of gram-negative bacteria. The gut microbiota disorder caused by obesity increases the abundance of some LPS-containing bacteria and leads to increased intestinal permeability; thus, LPS cannot pass through the intestinal mucosal barrier into the blood, inducing endotoxemia, a large number of pro-inflammatory responses, and the release of inflammatory cytokines. It is worth noting that the intestinal mucosal barrier of HFD-induced obese mice can be repaired after treatment with SZ-A, which is consistent with the observed increase in the abundance of Akk, suggesting that treatment with SZ-A can relieve low-grade inflammation, improve metabolic disorders, and reduce body weight by regulating the flora disturbance caused by an HFD. Akk plays an important role in the study of inflammatory bowel disease (IBD). In IBD patients, the amount of Akk decreased significantly, whereas the total amount of other intestinal mucus-degrading bacteria increased, suggesting that Akk has anti-inflammatory effects that other bacteria may not (Li et al., 2022). Previous studies have confirmed that Akk has the function of promoting goblet cell proliferation, and improving intestinal mucosa and intestinal inflammation, which is consistent with the results of H&E (Cani et al., 2022). Akk is a mucin-degrading bacteria in the intestine, which mainly colonize the outer mucus layer of the gastrointestinal tract. It takes the mucin of the gastrointestinal tract as the source of carbon and nitrogen for its growth. Its mucin consumption and goblet cell regeneration can achieve a dynamic balance, thus maintaining the stability of the mucus layer (Paone and Cani, 2020). Akk can accelerate intestinal epithelial regeneration, promote intestinal epithelial cells development, and maintain intestinal homeostasis. Akk-secreted vesicles can reduce the expression of Toll-like receptor 4, thus regulating the NF-κB pathway and reducing the secretion of pro-inflammatory factors IL-6 and IL-8 (Liu et al., 2022).

Metabolomics results showed that oral administration of SZ-A could significantly improve the metabolic disorders of obese mice induced by the HFD, in which eicosapentaenoic acid (EPA), docosatetraenoic acid (DHA), alpha-linolenic acid, and lipocholic acid (LCA), and other unsaturated fatty acids were significantly increased compared to the HFD group. Many studies have shown that unsaturated fatty acids can regulate the expression of inflammation-related proteins and NF-kB, reduce inflammation, improve insulin sensitivity, reduce hunger, and improve obesity. In addition to regulating the secretion and absorption of cholesterol, triglycerides, and fat-soluble vitamins, bile acids also function as signaling molecules to improve metabolism and play an anti-obesity role by activating the G-protein-coupled receptor 5 (TGR5) and Farnesoid X receptor (FXR). In conclusion, this study demonstrated that oral administration of SZ-A can improve the symptoms of metabolic disorders by regulating the gut microbiota of HFD mice.

## 5 Conclusion

In this study, the effects of SZ-A on regulating metabolic disorders and maintaining gut microbiota were investigated in HFD-induced obese mice. The results showed that SZ-A could improve the symptoms of obesity, in which body weight, total fat, serum total cholesterol, and low-density lipoprotein significantly decreased compared with the HFD group. After the treatment of SZ-A, the improvement of gut microbiota maintained the level of Firmicutes and *Bacteroides* at a reasonable level, and the increased abundance of Akkermansia muciniphila and Bifidobacterium also contributed to the weight loss of HFD-induced obese mice. Additionally, SZ-A regulates metabolic disorders in obese mice by regulating amino acids, unsaturated fatty acids, bile acids, and other metabolic pathways. In conclusion, SZ-A can improve the indications of obesity and metabolic disorders in obese mice by improving gut microbiota and metabolism, and is expected to be an innovative drug for the treatment of the metabolic syndrome.

## Data Availability

The original contributions presented in the study are included in the article/Supplementary Material, further inquiries can be directed to the corresponding authors.
