# Supplementary material for: Ramulus mori (Sangzhi) alkaloids regulates gut microbiota disorder and its metabolism profiles in obese mice induced by a high-fat diet
Source: Front Pharmacol. 2023 Mar 31;14:1166635. doi: 10.3389/fphar.2023.1166635 (PMC10102453; doi:10.3389/fphar.2023.1166635)
Supplement: Supplementary file 1 [file DataSheet1.PDF]

Ramulus Mori (Sangzhi) Alkaloids regulates gut microbiota disorder and its metabolism profiles in obese mice induced by a high-fat diet

Dongdong Liu[1,2†], Jun Ye[2†], Yu Yan[1,2], Yanmin Chen[2,3], Hongliang Wang[2], Mo Wang[2], Yu Feng[3], Renjie Li[2], Xiaoyan Xu[2], Yu Jiang[2], Chunfang Lian[2], Yanfang Yang[2], Yingying Meng[2], Yuling Liu[2*], Weizhe Jiang[1*]

[1]College of Pharmacy, Guangxi Medical University, Nanning 530021, P.R. China;

[2]State Key Laboratory of Bioactive Substance and Function of Natural Medicines, Institute of Materia Medica, Chinese Academy of Medical Sciences & Peking Union Medical College, Beijing 100050, P.R. China;

[3]Beijing Wehand-Bio Pharmaceutical Co., Ltd, Beijing 100260, P.R. China.

[†]Dongdong Liu and Jun Ye contributed equally to this work and share the first authorship.

***Correspondence:**

Prof. Yuling Liu, State Key Laboratory of Bioactive Substance and Function of Natural Medicines, Institute of Materia Medica, Chinese Academy of Medical Sciences & Peking Union Medical College, 1 Xiannongtan Street, Beijing 100050, P.R. China, Email ylliu@imm.ac.cn;

Prof. Weizhe Jiang, College of Pharmacy, Guangxi Medical University, Nanning 530021, P.R. China, Email jiangweizhe6812@163.com.

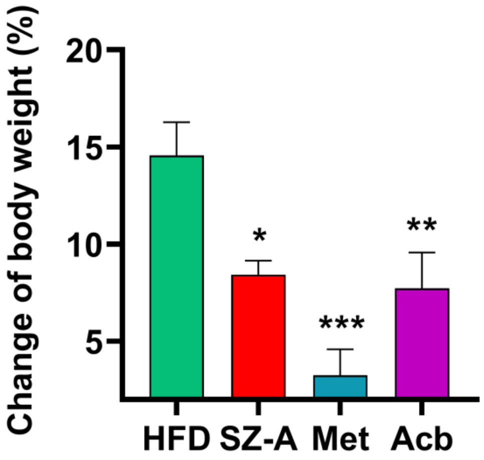

Figure S1. The change of body weight of mice treated with SZ-A (200 mg/kg), Met (300 mg/kg), and Acb (100 mg/kg). Each value is expressed as mean ± SEM. ***p < 0.001, **p < 0.01. *p < 0.05 compared with HFD.
